# The Pharmacopœia of the United States of America

**Published:** 1863-11

**Authors:** 


### Book Notices.

The Pharmacopœia of the United States of America. Fourth Decennial Revision. By Authority of the National Convention for Revising the Pharmacopœia, held at Washington, A.D., 1860. Philadelphia: J. B. Lippincott & Co. 1863.

This is a thoroughly revised, much improved, and neatly printed edition of the U. S. Pharmacopœia. Instead of attempting to enumerate the several important changes and additions made in this edition, we will simply say, that the Committee of Revision was composed of men thoroughly qualified for the duties assigned them, and they have performed their task well. The present volume is a small-sized octavo, containing about 400 pages, on good paper and excellent type. Price, $1.00. For sale by S. C. Griggs & Co., of this city.

